# Three-Dimensional CuO/TiO_2_ Hybrid Nanorod Arrays Prepared by Electrodeposition in AAO Membranes as an Excellent Fenton-Like Photocatalyst for Dye Degradation

**DOI:** 10.1186/s11671-020-3266-6

**Published:** 2020-02-18

**Authors:** Manisha Kondiba Date, Li-Heng Yang, Tzu-Yi Yang, Kuang-ye Wang, Teng-Yu Su, Ding-Chou Wu, Yu-Lun Cheuh

**Affiliations:** 1 Department of Materials Science and Engineering, National Tsing Hua University, Hsinchu, 30013 Taiwan; 2 Department of Physics, National Sun Yat-Sen University, Kaohsiung, 80424 Taiwan; 3 Frontier Research Center on Fundamental and Applied Sciences of Matters, National Tsing Hua University, Hsinchu, 30013 Taiwan

**Keywords:** Anodic aluminum oxide, Semiconductor nanorod array, Photo-Fenton-like reaction, Template-assisted electrodeposition, Dye photo-degradation

## Abstract

Three-dimensional (3D) CuO/TiO_2_ hybrid heterostructure nanorod arrays (NRs) with noble-metal-free composition, fabricated by template-assisted low-cost processes, were used as the photo-Fenton-like catalyst for dye degradation. Here, CuO NRs were deposited into anodic aluminum oxide templates by electrodeposition method annealed at various temperatures, followed by deposition of TiO_2_ thin films through E-gun evaporation, resulting in the formation of CuO/TiO_2_ p-n heterojunction. The distribution of elements and compositions of the CuO/TiO_2_ p-n heterojunction were analyzed by EDS mapping and EELS profiles, respectively. In the presence of H_2_O_2_, CuO/TiO_2_ hybrid structure performed more efficiently than CuO NRs for Rhodamine B degradation under the irradiation of 500-W mercury-xenon arc lamp. This study demonstrated the effect of length of CuO NRs, on the photo-degradation performance of CuO NRs as well as CuO/TiO_2_ heterostructure. The optimized CuO/TiO_2_ hybrid NR array structure exhibited the highest photo-degradation activity, and the mechanism and role of photo-Fenton acting as the catalyst in photo-degradation of dye was also investigated.

## Background

The industrial revolution of the 1760s made human life easier. However, industries generate toxic compounds and discharge serious contaminants, which are harmful for individuals and the environment. Especially in developing countries, the issue of environmental pollution is getting worse because of the growth in textile and petrochemical industries, which discharge organic waste into the water bodies. Thus, wastewater treatment has become a critical necessity [1, 2]. There are various methods for wastewater treatment, which can be classified into physical, chemical, and biological processes. The advanced chemical oxidation process (AOP) is one of the most stable and powerful methods, which facilitates the destruction or decomposition of organic molecules [3]. Generally, AOPs present a great degradation ability with the rapid generation of reactive hydroxyl radical (OH·), a harmless, powerful, and short-lived oxidant. In particular, the Fenton system, which has been well studied since the 19th century, is a good candidate for the removal of industrial organic contaminants [4, 5]. Fenton (Fe^2+^/H_2_O_2_) or Fenton-like (e.g., Fe_3_O_4_/H_2_O_2_) systems are widely used in organic pollutant degradation [6, 7]. Fenton-like catalysts, such as Fe-based materials are more stable, controllable, and harmless, exhibiting excellent efficiency as high as the Fenton catalyst. In some cases, they perform better in harsh environments, including inappropriate pH and in the presence of reactive substances in the solution, which may cause precipitation or absorption, resulting in the consumption of catalysts [8–10]. Apart from Fe-based materials, some Cu-based materials also show great performance in the Fenton-like system.

Furthermore, the catalysis performance can be reinforced by involving extra energy, such as heat, irradiation, electric, and vibration power [11]. Among them, the catalyzed photolysis, namely photocatalysis, has attracted much attention due to its simplicity and easiness. There are two important properties, which dominate the photocatalytic performance. One is the ability of the catalyst to create electron-hole pairs, which is associated with photocatalytic reaction to generate free radicals of water oxidizing reactants [12–15]. Another is the well-separation of electron-hole pairs generated through light emission, which prevent the recombination. Semiconductor materials are much suitable to act as a photocatalyst with their narrow bandgap, which makes it easy for the electrons to be excited from the valence band (VB) to the conduction band (CB) when absorbing optimum heat or luminous energy. One of the most widely used photocatalysts is titanium dioxide, which is an n-type metal oxide semiconductor and has been extensively studied due to its high activity and low cost [16–19]. In addition, copper oxide (CuO) is a great Fenton-like, narrow bandgap, and p-type metal oxide semiconductor photocatalyst.

The anodic aluminum oxide (AAO) is a self-assembled and ordered hexagonal honeycomb-like nano-porous structure with high-density arrays of uniform and parallel pores fabricated by an electrochemical etching method, which has been widely studied [20–26]. The diameter of the pores can be as low as a few nanometers and as high as several hundred nanometers, and length can be controlled from a few nanometers to more than hundreds of micrometers. The size of the porous structure can be correlated to different anodizing conditions, including electrolyte, voltage, and current density [27–38]. Additionally, pulsed current electroplating can precisely control deposition properties at room temperature, including deposition rate and the crystallinity by changing the step current and frequency [39–44]. Nonetheless, a relatively long relaxation between the pulses releases the stress during deposition, which can be considered as the advantage of controllable nucleation and well-separated growth [45–47]. Besides, combination of the short duty cycle and high frequency can decrease the surface cracks.

In this regard, with AAO as a sacrificial template and the combination of pulsed electrodeposition process and E-gun evaporation deposition method, highly efficient and mass-produced catalysts were obtained. Here, the CuO was deposited into a pre-fabricated AAO by pulsed electrodeposition. Eventually, TiO_2_ was deposited by E-gun evaporation. Then, we focused on the improvement of non-ionic Fenton-like photocatalyst with NR-array structure for application in dye degradation. Obviously, CuO and TiO_2_ were combined to behave as a p-n heterojunction photo-Fenton-like catalyst, for which the distribution of elements and composition of the p-n heterojunction was analyzed by EDS mapping and EELS profiles, respectively. Performances of CuO NRs and CuO/TiO_2_ hybrid structure for Rhodamine B degradation under the irradiation of 500-W mercury-xenon arc lamp were studies in comparison. The effect on different lengths of CuO NRs as well as different annealing temperatures of CuO and TiO_2_ on photo-degradation of rhodamine B was studied in detail.

## Methods Section

### Materials and Reagents

Aluminum foil (99.99%, GUV Team Int), copper(II) sulfate pentahydrate (99.99%, Sigma Aldrich), copper chloride (97%, Alfa Aesar), perchloric acid (75%, J T Baker), oxalic acid (99.5%, J T Baker), ethanol (99.5%, Sigma Aldrich), hydrochloric acid (30%, FLUKA), phosphoric acid (99.99%, Sigma Aldrich), sodium hydroxide (98%, Sigma Aldrich), hydrogen peroxide (30%, Sigma Aldrich), potassium dichromate (99%, Merck), epoxy 353ND (EPO-TEK), and trisodium 2-hydroxypropane-1, 2, 3-tricarboxylate (99%, Merck).

We focused on the improvement of photocatalyst with nanorod (NR)-array hybrid structure for application in dye degradation. For the fabrication of highly efficient photocatalyst, copper oxide nanorods/titanium dioxide (CuO/TiO_2_) hybrid structure, template-assisted approach was used in combination with pulsed electrodeposition process and E-gun evaporation deposition method. For the formation of p-n heterojunction photocatalyst, the copper oxide (CuO) was deposited into the anodic aluminum oxide (AAO) by pulsed electrodeposition then titanium dioxide (TiO_2_) was deposited on top of it by E-gun evaporation. The effect on different lengths of CuO NRs as well as different annealing temperatures of CuO NRs and CuO/TiO_2_ hybrid structure on photo-degradation of rhodamine B were studied in detail.

### Formation of Anodic Aluminum Oxide (AAO)

Aluminum foil with the purity of 99.997% was procured form GUV Team International Co., Ltd. The Al foil was cut into equal shapes of 1 cm^2^ and flattened before electrochemical polishing at 40 V for 5~10 s in an electrolyte, which contained 20 vol.% perchloric acid and 80 vol.% absolute alcohol. The substrate was then rinsed with deionized water prior to use in anodization. The homemade AAO membranes were fabricated by a very well-known two-step anodization method. The first-step anodization was conducted in 0.3 M oxalic acid at 40 V for 10 min. The regularity ratio of AAO exhibited the maximum value, corresponding to minimum defects [31]. To control the stable growth of AAO, the solution was maintained at 10 °C by using the cooling system. Then, it was immersed in a solution of 2.24 wt.% potassium dichromate and 6 wt.% phosphoric acid at 60 °C for 1 h. The AAO was etched, leaving concaves on the surface of the substrate, which became the formation site for the growth during the anodic treatment. The second step, anodization for 20 min and 80 min, resulted in 1.85 μm and 6.53 μm channel length of AAO, respectively. After anodization was completed, the anodizing voltage was decreased to 5 V by altering the current stepwise within the current in the period of 5 min to reduce the thickness of the barrier layer. Through the barrier-thinning process the templates were made suitable for electrodeposition. Then, it was immersed into 5 wt.% phosphoric acid for 45 min at room temperature to widen the diameter of channels.

### Fabrication of Copper Oxide/Titanium Dioxide (CuO/TiO_2_) Hybrid Structure

Copper oxide (CuO) was deposited into anodic aluminum oxide (AAO) membrane by a well-known pulse electrodeposition method. The electrolyte contained 0.6 M copper sulfate, 6 wt.% trisodium 2-hydroxypropane-1, 2, 3-tricarboxylate and 10 μl of surfactant dissolved in 100 ml deionized (DI) water at room temperature. Non-symmetrical rectangular current, with pulses of 40 mA/10 ms and 0 mA/40 ms was supplied for the working electrode in a conventional three-electrode electrochemical cell. The pulses were applied in 6000 and 20,000 cycles for the AAO with two different lengths of 1.85 μm and 6.53 μm, respectively. After CuO deposition, annealing was performed in a tube furnace for 12 h at different temperatures of 400, 500 and 600 °C, in the presence of oxygen. In order to obtain fully oxidized copper oxide NRs, the O_2_ flux was maintained at 100 sccm. TiO_2_ with a thickness of 100 nm was deposited on the top of CuO/AAO by E-gun evaporation which covered the NR-array at the end of NRs. The second annealing of the sample was done at different temperatures of 400, 500 and 600 °C in a tube furnace for 5 h in oxygen ambient atmosphere. To increase the crystallinity and adhesion between two different metal oxides at the interface, the oxygen flux was kept 100 sccm. For transferring the catalytic film from the aluminum substrate to glass, the top side of (TiO_2_ side) sample was adhered to glass by using epoxy 353ND (EPO-TEK®) heated at 100 °C for 3 h. The transferred sample on the glass was then immersed in a solution consisting of hydrochloric acid, cupric chloride anhydrous, and DI water to remove the aluminum substrate through oxidation and reduction reaction between Al and Cu^2+^. Though aluminum was replaced by copper, the attachment of copper on the substrate was worse, with the remaining nanostructure covered by AAO. The residual aluminum oxide was removed by soaking the sample in 1 M sodium hydroxide solution for 5 h at room temperature.

### Dye Degradation of Copper Oxide/Titanium Dioxide (CuO/TiO_2_) Hybrid Structure

The titanium oxide thin film-capped CuO-nanorod (NR) arrays act as a substrate-assisted heterogeneous photo-Fenton-catalyst. Photo-Fenton-like reagents for degradation tests were prepared by adding an appropriate amount of catalyst to a 100-mL solution containing 50 ppm rhodamine B and 88 mM hydrogen peroxide, under a 500-W mercury-xenon arc lamp. The distance between the light source and solution was maintained at 20 cm. Prior to irradiation, the solution and catalyst were placed in the dark for 1 h to make sure that an adsorption/desorption equilibrium was established. Sampling was conducted at regular intervals of 5 min. Every time, a 100-μL solution was collected and then diluted into 10 mL deionized water before ultraviolet visible region spectroscopy (UV-Vis) measurements. The CuO NRs samples with a size of 1 cm^2^ were used during all degradation experiments. Initially, photo-degradation experiments were carried out with 1 mg of 1.85 μm long CuO NRs under different annealing temperatures of 400, 500, and 600 °C. The next set of experiments was performed with 1, 2, 3, and 5 mg of 1.85-μm-long CuO NRs annealed at 600 °C. Further, dye degradation measurements were conducted with 1 mg of 1.85-μm-long CuO NRs annealed at 600 °C combined with 100-nm-thick TiO_2_ annealed at 400, 500, and 600 °C. Then, photo-degradation measurements were executed with 6.53 μm (3 mg) and 1.85 μm (1 mg) long CuO NRs gathered with 100-nm-thick TiO_2_ annealed at 500 °C. A further set of measurements were performed with 100, 200, and 300-nm-thick TiO_2_ layers capping on 1.85-μm-long CuO NRs. The final set of photo-degradation measurements were carried out with the optimized catalyst: 1 mg of 1.85-μm-long CuO NRs (annealed at 600 °C) with 100-nm-thick TiO_2_ (annealed at 500 °C) added in 100 ml of 50, 250, and 750 ppm rhodamine B solution.

### Characterization

Surface morphologies and the lengths of NRs were confirmed by field-emission scanning electron microscopes (FE-SEM, Hitachi-SU8010). The bonding type and composition of materials (copper oxide (CuO) and titanium oxide (TiO_2_)) were verified by Raman spectroscopy analysis (HORIBA Jobin-Yvon, LabRAM, HR 800) equipped with a 532-nm laser. Phase and crystallinity results of the materials (copper oxide and titanium oxide) were collected by X-ray diffraction (D2 phaser, Cu Kα, *λ* = 0.154 nm) scanning in the 2θ ranging from 20° to 80°. The morphology, d spacings, and composition of TiO_2_-capped CuO NRs were determined by transmission electron microscope (TEM) with energy-dispersive x-ray spectroscopy (EDX) and electron energy loss spectroscopy (EELS). The degradation efficiency was calculated from the absorption data of rhodamine B measured by UV-visible NIR spectrophotometer (U-4100). Prior to TEM observation, the sample was cut into slices in nano-scale by focus ion beam technique. A thickness of slice under 50 nm is normally appropriate for TEM analysis, which provides a clear image and enables the EELS spectrum analysis.

## Results and Discussion

**Fig. 1:**
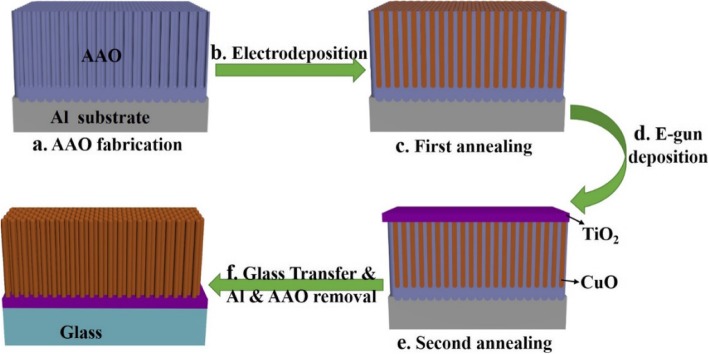
Schematic of the process flow for fabrication of CuO/TiO_2_ hybrid nanorod (NR) array

The heterogeneous photo-Fenton-catalyst fabricated in this work consists of two kinds of metal oxide semiconductors, including titanium oxide thin-film layer on copper oxide NR arrays. The overall process is schematically illustrated in Fig. 1. The AAO with two different lengths of 1.85 μm and 6.53 μm were fabricated on an aluminum substrate, using a two-step anodization process followed by barrier thinning. For the formation of CuO NRs, copper oxide (CuO) was deposited into AAO membrane by pulse electrodeposition with the controlled number of cycles. To obtain fully oxidized copper oxide NRs, the first annealing of samples was performed at varying temperatures for 12 h under an O_2_ ambient. The deposition of TiO_2_ was then carried out by E-gun evaporation to form a thin film with a thickness of 100 nm on the top of CuO/AAO. In order to increase the crystallinity and adhesion between two different metal oxides at the interface, the second annealing of samples was performed at 400, 500, and 600 °C for 5 h under the O_2_ ambient. For further process, the catalytic film was then transferred from aluminum substrate to glass. The aluminum substrate was removed first; then, residual aluminum oxide was removed from the substrate. The final glass samples were further used for characterization and measurements.

**Fig. 2:**
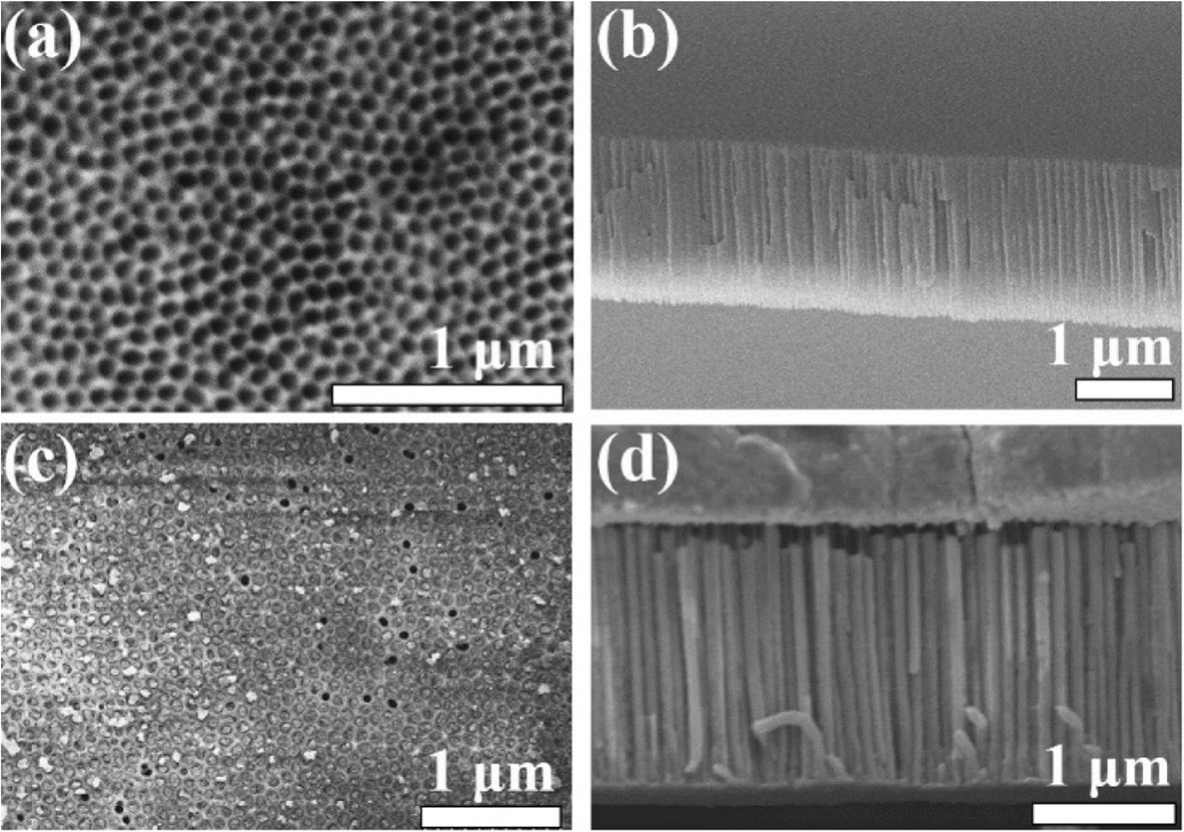
**a** Top view and **b** cross-section SEM images of AAO before electrodeposition. **c** Top view and **d** cross-section view SEM images of AAO after CuO electrodeposition (scale bar, 1 μm)

The morphology of AAO and template-assisted CuO NR arrays were observed by FE-SEM as shown in Fig. 2. Figures 2 a and b show top view and cross-section view FE-SEM images of AAO, respectively, with which the AAO with an average pore diameter of ~ 76 nm and length of ~ 1.85 μm were confirmed. Figures 2 c and d show top view and cross-section view SEM images of CuO NRs inside AAO where CuO NRs were prepared using AAO with a channel length of 1.85 μm. From Figs. 2 c and d, the CuO NRs were well-deposited in the AAO with a high filling rate by electrodeposition. Similarly, CuO NRs with lengths of 6.53 μm were prepared using AAO with a channel length of 6.53 μm confirmed from a cross-section view SEM image as shown in Additional file 1: Figure S1. The AAO template-assisted technique can ensure repeatability for the fabrication of CuO NRs.

**Fig. 3:**
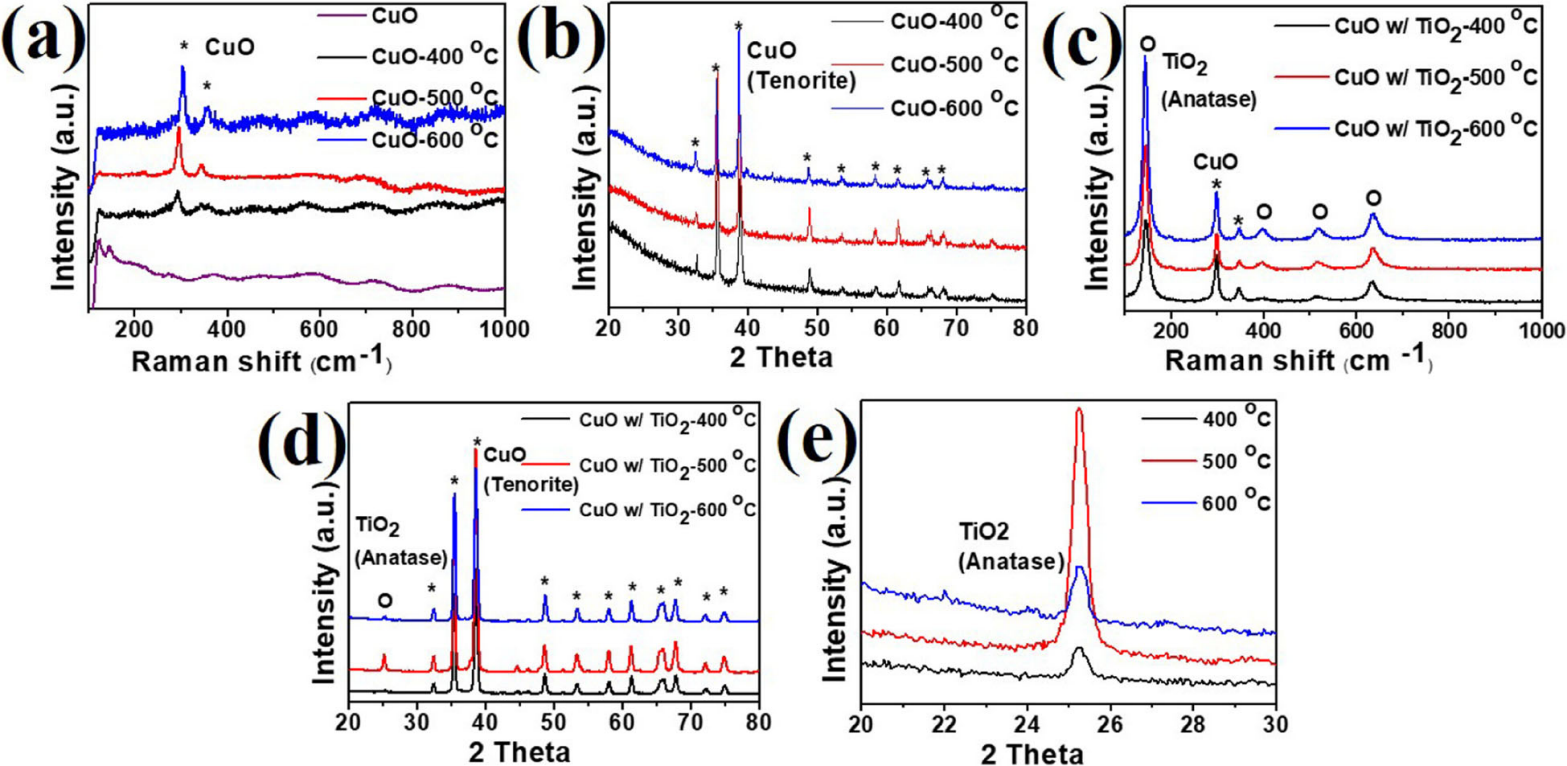
**a** Raman and **b** XRD results of CuO NRs annealed at different temperatures. **c** Raman and **d** XRD results of CuO/TiO_2_ annealed at different temperatures. **e** The magnified view of the XRD results for CuO/TiO_2_ at 2θ ranges of 20–30°

The crystallinity and composition of materials were verified by Raman and XRD results, which indicated the quality and phase of the material. For Raman and XRD analysis, the samples were transferred to the glass substrate, followed by the removal of Al substrate and AAO. More details of the above process are mentioned in the experimental section. Following the above process, a total of 7 samples were prepared for Raman and XRD analysis, including raw CuO NRs, CuO NRs annealed at different temperatures, and CuO NRs/TiO_2_ structure annealed at different temperatures. The Raman shifts of CuO_1-x_ NRs prepared under different annealing temperatures from 400, 500, and 600 °C are shown in Fig. 3 a. Two peaks at 297 cm^−1^ and 352 cm^−1^ can be found in Raman spectra for CuO_1-x_ NRs after the annealing processes, matching well with the standard pure CuO with the tenorite phase. The results of Raman analysis were corroborated by the XRD analysis. The observed peaks in XRD analysis are 32.5°, 35.5°, 38.7°, 48.7°, 58.3°, and 61.5° in 2θ, corresponding to (110), (11$\overline{1}$), (111), (20$\overline{2}$), (202), and (11$\overline{3}$) planes, respectively in tenorite phase as shown in Fig. 3 b. The CuO NRs in AAO templates completely oxidized and transformed to tenorite phase under the high annealing temperature over 400 °C held for 12 h in an oxygen ambient. Besides, with the higher annealing temperature, the crystallinity increases, which was proved by calculating the full width at half maximum (FWHM) of the main peaks in the tenorite phase. By Gaussian function fitting, the FWHMs of the (11$\overline{1}$) peak for CuO samples annealed at 400, 500, and 600 °C corresponds to 0.284°, 0.251°, and 0.22°, respectively. The The FWHM decreases as the annealing temperature increases, revealing improvement of crystallinity and grain growth. Furthermore, the crystal structure of E-gun-deposited TiO_2_ thin film covering CuO under different annealing temperatures is shown in Fig. 3 c. Raman spectrum showed that pure CuO and anatase phase of TiO_2_ achieved after the annealing temperatures of 400, 500, and 600 °C. The Raman peaks at 145, 397 [1], 516, and 637 cm^−1^ represent the anatase phase of TiO_2_ while peaks at 299 and 397 cm^−1^ depict pure CuO. In XRD results for CuO/TiO_2_ as shown in Fig. 3 d, the peak at 2θ = 25.3° shows the existence of the anatase phase of TiO_2_ in (101) plane while the other peaks were contributed from the existence of CuO. Distinctly, the crystallinity of the anatase TiO_2_ phase increases as the annealing temperature increases from 400 to 500 °C. However, it decreases upon raising the temperature further from 500 to 600 °C as confirmed by FWHM results. Based on the enlarged view of the diffraction peak related to the (101) plane, the FWHMs of 0.432, 0.411, and 0.416° in 2θ were calculated for the annealing temperatures of 400, 500, and 600 °C, respectively, as shown in Fig. 3 e. The decrease of crystallinity of anatase TiO_2_ was related to the nucleation of the rutile phase at the phase transition temperature of 600 °C [48]. However, Raman analysis did not show the rutile phase, which is usually obtained at 600 °C. Nevertheless, Additional file 1: Figure S2 reveals the existence of the rutile phase by XRD analysis of TiO_2_ over the 2θ ranges of 25–29°.

**Fig. 4:**
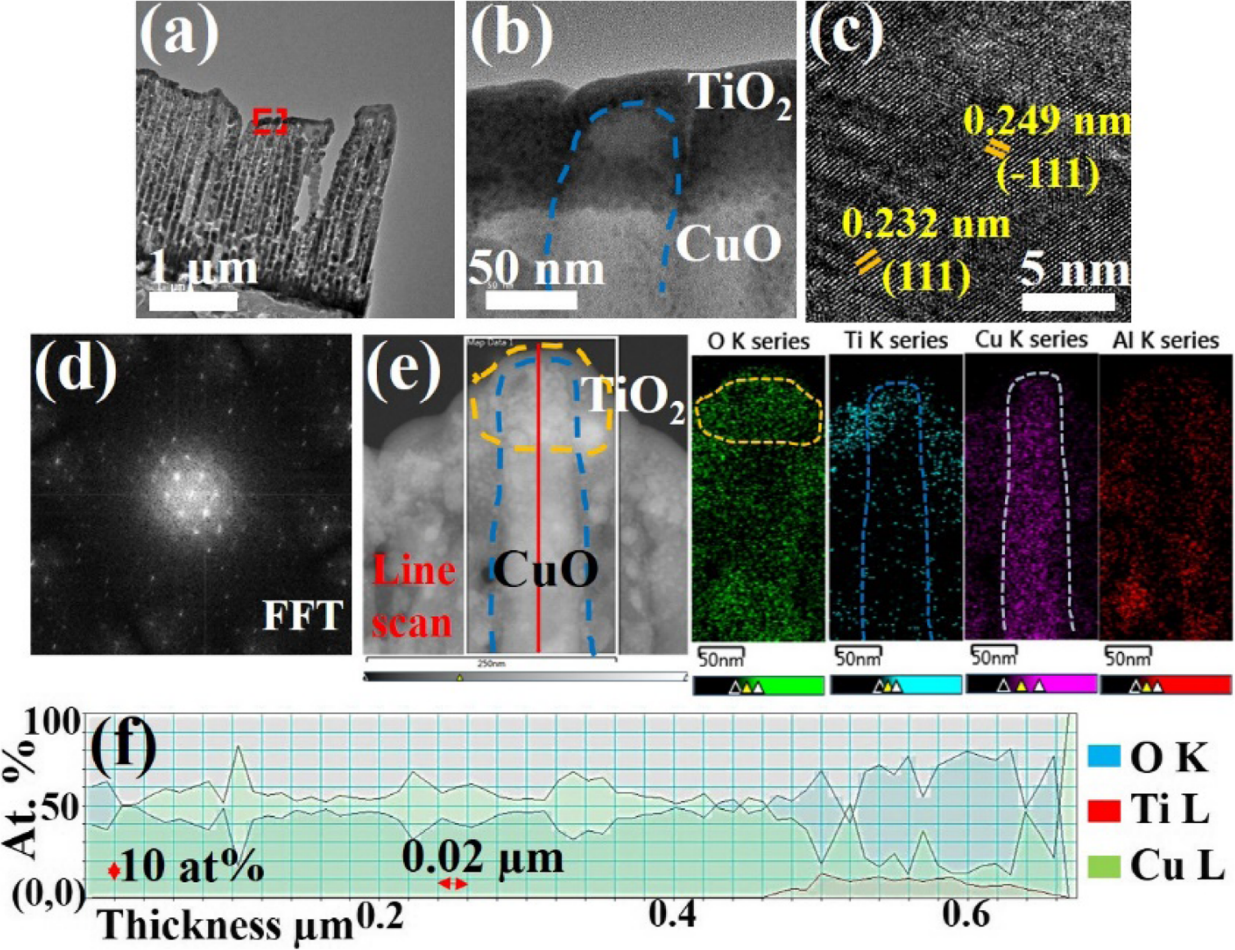
**a** A low-magnification TEM image of CuO NRs array capped by a TiO_2_ thin layer. **b** The corresponding HRTEM image of the TiO_2_-capped CuO NR taken from the rectangular area indicated in **a**. **c** d-spacing and **d** FFT results of CuO NRs. **e** EDS mapping images and **f** EELS line scan results of CuO/TiO_2_

Typical low-magnification image of TiO_2_ thin film-coated CuO NRs array, which was annealed at two stages, the first annealing process was conducted at 600 °C for 12 h after the CuO deposition and the second annealing process was conducted at 500 °C for 5 h after the TiO_2_ deposition as shown in Fig. 4 a. Figure 4 b shows the high-resolution TEM (HRTEM) image of the selected part from Fig. 4 a, with which the TiO_2_ thin film is well-deposited on the top of the CuO NRs. As can be seen in Fig. 4 b, the clarified TiO_2_ layer coated on the top of CuO NRs can be confirmed. The d spacing calculated by FFT and the FFT images of CuO NRs are shown in Figs. 4 c and d, respectively. The CuO exhibits the d spacings of 0.232 nm for (111) plane and 0.249 nm for$\Big(\overline{1}11$), respectively. The lattice constants and diffraction patterns match well with the tenorite phase of CuO (JCPDS card #05-0661). Figure 4 e shows the EDS mapping images of the TiO_2_-capped CuO NRs. Components mapping images from EDS results indicate the uniform distribution of elements and the titanium signal concentrated in a local area at the top of CuO NRs in a mushroom-like shape can be found. EELS profiles as shown in Fig. 4 f reveal the compositions of titanium, oxygen, and copper, respectively. Titanium signal is present only at one side while copper and oxygen signals appear through the whole structure but in different ratios between covered and non-covered regions. The Cu and O signals are well-distributed with a ratio of nearly 1:1 in CuO NRs whereas Cu:O:Ti signals at the covered region show a ratio of 3:6:1, respectively.

For the purification of dye effluents and wastewater treatment, the photo-degradation of Rhodamine B (RhB) has been intensively studied [49, 50]. It is both highly water and organic soluble, the basic red dye of the xanthene class, which has been found to be potentially toxic and carcinogenic, is widely used as a colorant in textiles and foodstuffs. It is also a well-known water tracer fluorescent dye [51, 52]. The absorbance in relation to the color change caused by decolorization can be determined by measurements of UV-vis results. The absorbance was recorded in wavelength ranging from 450 to 600 nm in the red light region and the RhB showed a maxima result for the light absorption at 554 nm. The absorbance of light-absorbing material is proportional to its concentration according to the following equation:

1 $$ \mathrm{A}=\log \left(\frac{I}{I_o}\right)=\log \left(\frac{1}{T}\right)=\upalpha \mathrm{lc} $$

2 $$ \frac{\mathrm{C}}{C_o}=\frac{\mathrm{A}}{A_o} $$

Where *A*_*o*_ and *A* are the absorbance of the dye solution before and after irradiation, *I* and *I*_*o*_ are the intensity of incident and transmitted light, *T* is the transmittance of light, *α* is the absorption coefficient, *l* is the length of path of sample, and *C*_*o*_ and *C* are the concentration of dye solution before and after irradiation, respectively. The efficiency of the photo-degradation can be measured by the relation between concentration and absorbance in an appropriate wavelength range [53]. However, at the high concentration, the concentration to absorbance curve does not follow the equation because of the non-linear behavior. On the other hand, at lower dye concentrations, a considerable part of hydroxyl and hydroperoxyl radicals recombines to yield H_2_O_2_ and the degradation was carried out in a lower concentration of OH radicals. The excess oxygen bubbles absorb the free radicals, leading to the decrease of reagents as only ~ 10% of the OH radicals generated in the bubble can diffuse into the solution, thereby causing a low degradation rate. With the increase in the dye concentration, the degradation rate rises and meets the equilibrium condition when it reaches a saturation limit. We calculated the ratio between absorbance and concentration under different degradation time and then obtained the degradation rate under various operating conditions. Furthermore, the information on the concentration variation indicates the order of a chemical reaction. Usually, for the dye decomposition, the reaction is a pseudo-first-order reaction. The equation for calculating the order of the reaction is shown below:

3 $$ \mathrm{C}={C}_ot+B $$

4 $$ \ln \left(\frac{\mathrm{C}}{C_o}\right)= kt+B $$

5 $$ \frac{1}{\mathrm{C}}=\frac{1}{C_o}+ kt $$

Where *C* is the concentration, *t* is reaction time, *k* is equilibrium constant, and *B* is a constant. The photocatalytic activity was revealed by measuring the degradation rate of RhB solution under different conditions. Note that Equation (3) represents zero-order reaction while Equations (4) and (5) represent first-order and second-order reactions, respectively. The concentration profile indicates not only the activity but also the reaction order. Here, we measured the reaction order by changing the dosage of the catalyst. The system can be classified as the pseudo-first-order reaction. The degradation rate increases with the increase in dosages and meets an equilibrium condition owing to the saturation of reactants attached to the interface of catalyst/solution. It happened because the surface area for the heterogeneous catalyst is one of the determining factors of the reaction. With a larger surface area to mass ratio, the required dosage of the catalyst to reach equilibrium condition became much less. In our case, for an equilibrium condition, approximately 3 mg dosage is needed, and then the kinetic equilibrium constant *k* can be calculated as 0.436 min^−1^.

**Fig. 5:**
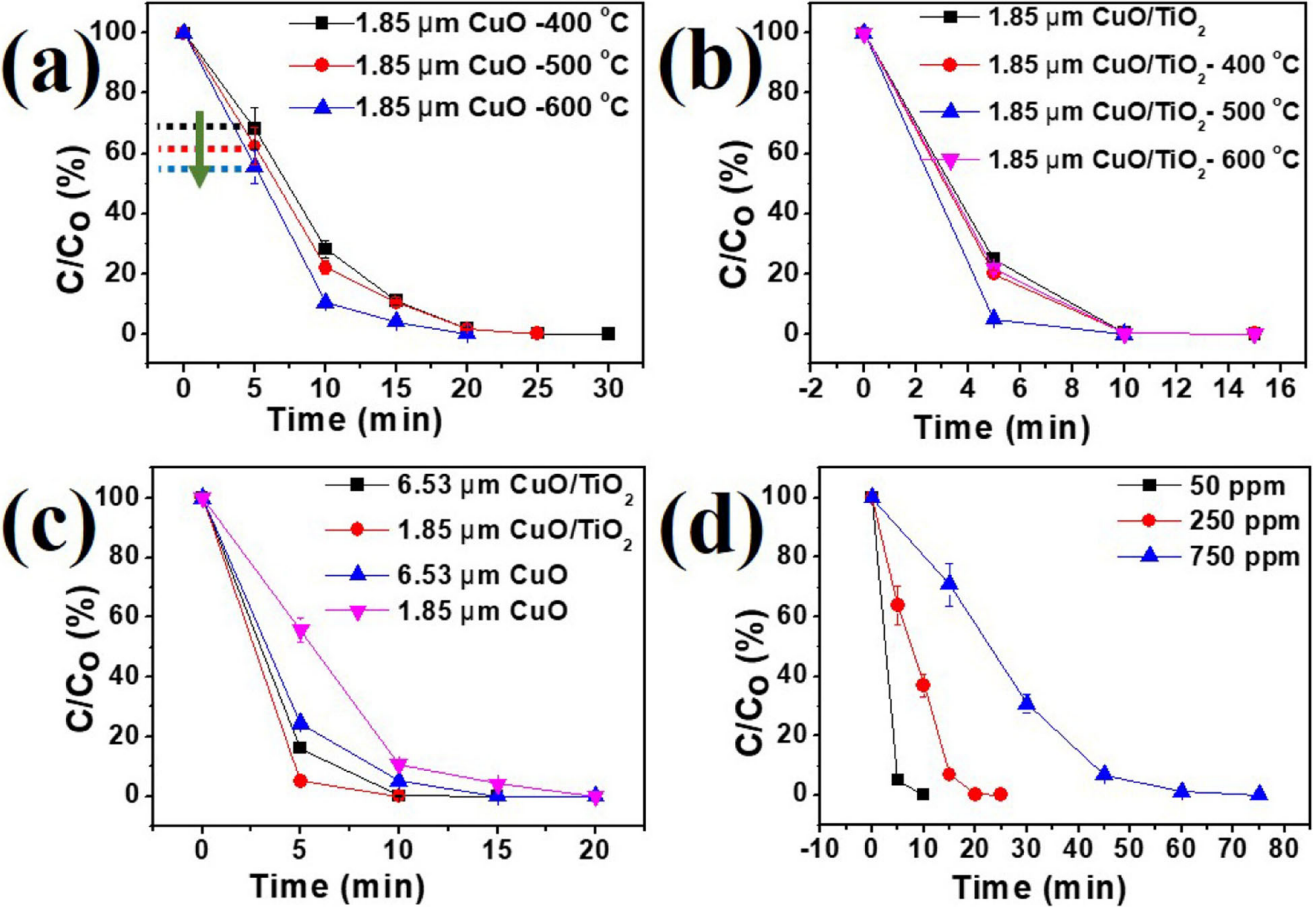
Degradation results of **a** CuO NRs samples annealed at different temperatures. **b** CuO/TiO_2_ samples annealed at different temperatures. **c** Samples at different lengths of CuO NRs with and without capping of the TiO_2_ layer. **d** Different initial concentrations of RhB with the most active sample (600 °C 1.85 μm CuO NRs + 500 °C TiO_2_)

**Fig. 6:**
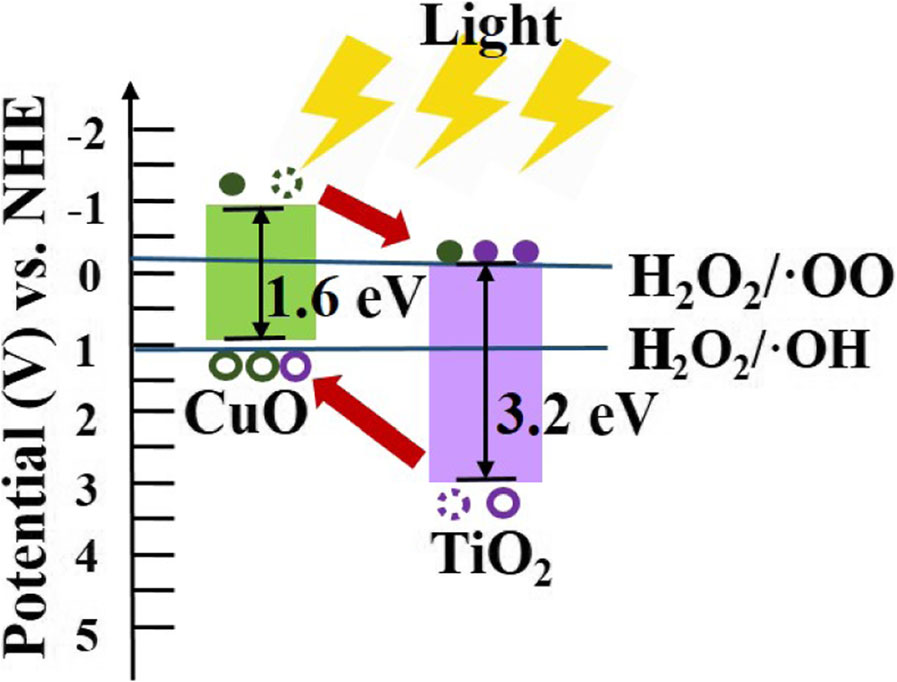
Band diagram of CuO and TiO_2_ at pH = 7 [55, 56]

Figure 5 a shows the photocatalytic performance of 1 mg CuO NRs with a length of 1.85 μm under different annealing temperatures of 400, 500, and 600 °C for 12 h in the oxygen ambient. The increasing annealing temperature to 600 °C results in the higher crystallinity of the catalyst, which exhibits better performance. Degradation rates of the RhB using TiO_2_-capped CuO NRs annealed at different temperatures of 400, 500, and 600 °C for 5 h in the oxygen ambient are shown in Fig. 5 b. With the anatase TiO_2_-capped CuO NRs, the catalyst shows excellent efficiency. Besides, the photocatalytic activity can be further improved after the annealing treatment. Interestingly, the sample annealed at a temperature of 500 °C shows the best photocatalytic activity while the sample annealed at 600 °C exhibits a decreased photocatalytic performance. As a result, the CuO/TiO_2_ hybrid NR array annealed at 500 °C demonstrated the highest catalytic performance, yielding a kinetic equilibrium constant *k* of 0.921 min^−1^. The reason why the catalyst annealed at 600 °C showed the lower performance than 500 °C is related to the presence of the rutile phase. Under the O_2_ ambient condition, the phase transformation of TiO_2_ from anatase to rutile phase occurs at a temperature of ~ 600 °C (Additional file 1: Figure S2) [48]. When the annealing temperature reached the phase transformation temperature, the photocatalytic activity of TiO_2_ decreases due to the formation of the nucleation to the rutile phase. Generally, TiO_2_ composed of mixed-phase with a certain ratio between anatase and rutile phase exhibits better conductivity and photocatalytic property than a single phase of both anatase and rutile phase. In this case, the annealing condition for TiO_2_ underwent the phase transformation temperature. As the nucleation of the rutile phase reduces the grain size of the anatase phase, the crystallinity of TiO_2_ with the rutile phase decreases, resulting in poor photocatalytic activity. The effect of two different lengths of CuO NRs in CuO/TiO_2_ on photo-degradation performance is shown in Fig. 5 c. For only CuO NR samples, the longer length of NRs (6.53 μm) contributed to the larger dosage of the catalyst, which exhibited the better photocatalytic performance than that of shorter length NRs. For the CuO NRs combined with TiO2 thin film, the penetration depth of the light may play an important role. Only when the depletion zone is exposed to irradiation, the p-n heterojunction semiconductor presents an excellent photo-activity. Then, the photo-excited electron-hole pairs can rapidly separate and react with reagents. Here, the penetration depth can be calculated by the following equation, *d* = 1/*α*, where *α* represents the absorption coefficient of the CuO. The distribution of the spectrum of mercury-xenon arc lamp is near UV light with photon energy over 3 eV. According to different axes of the CuO, the calculated penetration depth from the simulation results in 1~5 μm [54]. Hence, the CuO NRs with a length of 1.85 μm exhibited excellent performance for the heterostructure. In addition, the effect on lengths of NRs in CuO NRs and CuO/TiO_2_ associated with the penetration depths of the incident light are shown in Fig. 5 c. Note that the longer length of NRs (6.53 μm) in heterostructure restricts the light to reach the depletion zone. Thus, CuO NRs with a length of 1.85 μm covered by the TiO_2_ layer exhibit a much better catalysis effect compared with that of CuO NRs with a length of 6.53 μm covered by the TiO_2_ layer. The measurements on the degradation of RhB were conducted under differently initial RhB concentrations with the most active sample, namely CuO NRs with a length of 1.85 μm annealed at 600 °C after combining with the TiO_2_ layer annealed at 500 °C as shown in Fig. 5 d. For initial RhB dosages of 50, 250, and 750 ppm, the reaction completed in 10, 25, and 75 min, respectively. The band diagram of CuO/TiO_2_ is a staggered gap (type II) heterojunction semiconductor as shown in Fig. 6.

The basic principle of photo-Fenton-catalysis is an oxidation and reduction reaction referring to contaminants decomposed by hydroxyl and hydroperoxyl radicals, which are produced by H_2_O_2_ with the help of catalyst through the excited electron-hole pairs under the irradiation [50, 57, 58]. Note that the reaction of the pseudo-first-order reaction has been confirmed according to the degradation rate-dosage profile, which is a common type for a heterogeneous catalyst [59]. Although the larger surface area contributed from more dosages of the catalyst provides regions for H_2_O_2_ to attach on the interface, an equilibrium concentration of hydroxyl and hydroperoxyl radicals can be related to the kinetics at various conditions, such as the temperature, irradiation, and pH. With enough attachment of H_2_O_2_, the reaction seemed nearly first-order, which means the chemical reaction acted as the rate-determining step and not the diffusion. The reactions for the decomposition of H_2_O_2_ are shown below.

6 $$ \mathrm{CuO}\left({\mathrm{h}}^{+}-{\mathrm{e}}^{-}\right)+{\mathrm{H}}_2{\mathrm{O}}_2=\mathrm{OH}\cdotp +{\mathrm{O}\mathrm{H}}^{-}+\mathrm{HOO}\cdotp +{\mathrm{H}}^{+} $$

7 $$ \mathrm{CuO}\left({\mathrm{h}}^{+}\right)-{\mathrm{TiO}}_2\left({\mathrm{e}}^{-}\right)+{\mathrm{H}}_2{\mathrm{O}}_2=\mathrm{OH}\cdotp +{\mathrm{O}\mathrm{H}}^{-}+\mathrm{HOO}\cdotp +{\mathrm{H}}^{+} $$

8 $$ \mathrm{RhB}+\mathrm{OH}\cdotp +\mathrm{HOO}\cdotp =\mathrm{Oxidized}\ \mathrm{product} $$

The excited electrons react with H_2_O_2_, producing OH· radical while electron-holes oxidize H_2_O_2_, generating HOO· radical. As deduced from the equation, the more electron-hole pairs are generated, the more radicals are involved in the system, which eventually raises the degradation rate. For the photo-Fenton-like heterogeneous catalyst, CuO NR arrays promote the reaction by its electron-hole pairs generated upon the irradiation. A cross-linked region in the energy level of CuO and H_2_O_2_ exhibited the tendency for the electron-hole pairs in CB while the VB attracted the H_2_O_2_ producing HOO· and OH· radicals, respectively. An alternative reaction mechanism generated through the involvement of catalyst with lower activation energy referred to larger kinetic constant *k*, which became a rate-determining factor of the chemical reaction. The change of band profile leads to a reinforced phenomenon of the separation of the electron-hole pairs, which made the lifetime of electron-hole pairs longer for the reaction. Among different phases of TiO_2_, the anatase phase is much suitable to be applied in the heterojunction as the indirect bandgap of the anatase phase exhibits a longer lifetime of photo-excited electrons and holes than the direct bandgap of rutile and brookite phases. Also, the effective mass of photo-generated electrons and holes were the lightest, which contributed to better current transportation with higher performance [60]. This is the reason why the degradation rate decreases when the rutile phase appears. The increase in the thickness of TiO_2_ thin film does not influence the photo-degradation performance as shown in Additional file 1: Figure S3 where only 100-nm-thick TiO_2_ thin film is thick enough to form a well-developed depletion zone of p-n heterojunction. Furthermore, the comparison between different catalysts for dye degradation is shown in Table 1 where our catalyst shows the superior photocatalytic performance with a small dosage of CuO/TiO_2_ NR array heterostructure.

**Table 1:** Comparison table of different catalysts from earlier researches with our work for dye degradation

Sr No.	Catalyst	Dosage	H_2_O_2_	RhB	pH	Time	Power	Ref.
1	Pyrite	50 mg	6 mM	20 ppm 50 ml	3	120 min	-	[10]
2	CuO	20 mg	176 mM	200 ppm 100 ml	-	5 h	-	[61]
3	CuO/Ag	20 mg	2.94 M	5000 ppm 50 ml	5	60 min for 30%	150 W	[62]
4	CuO /graphene	20 mg	4 mM	5 ppm 50ml	-	15 min	500 W	[63]
5	Fe_3_O_4_	50 mg	40 mM	5 ppm 50 ml	6.4	120 min	-	[7]
6	LaFeO_3_	50 mg	6 mM	10 ppm 100 ml	4	30 min	-	[64]
7	Fe–Si–B amorphous ribbons	100 mg	1.6 mM	20 ppm 200 ml	3	10 min	-	[65]
			0.5 M		neutral	60 min for 70%		
8	Ag_3_PO_4_-Bi_2_MoO_6_	50 mg	17.6 mM	10 ppm 50 ml	-	100 min	300 W	[66]
9	Co_x_Mn_3-x_O_4_	10 g	5.88 mM	30 ppm 500 ml	6.29	80 min	-	[67]
10	CuO/LaFeO_3_	15 mg	147 mM	30 ppm 100 ml	-	120 min	300 W	[68]
11	rs-TNSs	50 mg	100 mM	20 ppm 50 ml	-	5 min	300 W	[69]
12	Fe_2_O_3_–Kaolin	100 mg	147 mM	15 ppm 100 ml	2.21	40 min	300 W	[70]
13	Graphene oxide–Fe_2_O_3_	100 mg	147 mM	100 ppm 100 ml	2.09	80 min	300 W	[71]
14	Zn-doped Fe_3_O_4_	100 mg	8.8 mM	10 ppm 100 ml		180 min	350 W	[72]
15	SnO_2_-encapsulated -Fe_2_O_3_ nanocubes	15 mg	400 mM	25 ppm 25 ml	7.33	60 min	19.6 mW/cm^2^	[73]
16	Fe^II^–Co PBA	200 mg	4 mM	12 ppm 40 ml	4.8	30 min	13.5 mW/cm^2^	[74]
17	Cu_2_(OH)PO_4_/g-C_3_N_4_	200 mg	176 mM	10 ppm 50 ml	5	40 min	500 W	[75]
18	CuO nano-rods + TiO_2_	1 mg	88 mM	50 ppm 100 ml	neutral	10 min	500 W	Our work
			265 mM	250 ppm 100 ml		25 min		
				750 ppm 100 ml		75 min		

## Conclusions

In summary, high-aspect ratio TiO_2_ thin film-capped CuO NR arrays synthesized by utilizing e-gun evaporation deposition and electrodeposition in the AAO template exhibited great photo-Fenton-like catalytic properties. CuO NRs with tenorite phase was obtained after annealing over 400 °C for 5 h. The anatase phase of the TiO_2_ thin film after annealing at 400 °C for 12 h can be formed while the rutile phase occurs with the annealing temperature at 600 °C for 12 h. For CuO NRs, NRs with a length of 6.53 μm exhibited higher efficiency, which could be attributed to a larger amount of catalyst dosages. Also, the higher crystallinity of CuO NRs obtained by raising in annealing temperature leads to the higher photocatalytic activity. However, the presence of the rutile phase of TiO_2_ under higher annealing temperature decreased the photocatalytic performance. In addition, the shorter length of CuO NRs (1.85 μm) in CuO/TiO_2_ heterojunction exhibited better performance due to the shorter penetration depth of UV light. With an increase in the thickness of TiO_2_ thin film in CuO/TiO_2_ heterojunction, the degradation performance remained uninfluenced.

## Supplementary information

**Additional file 1: Figure S1.** Cross-section SEM image of 6.53 μm long CuO NRs in AAO (scale bar: 1 μm). **Figure S2.** XRD spectrum of TiO_2_ capping CuO NRs annealed at 600°C, over the 2θ ranges of 25°-29°. **Figure S3.** Degradation results of different TiO_2_ thickness annealed at 500 °C capping 1.85 μm long CuO NR arrays.

## Data Availability

All data generated or analyzed during this study are included in this published article and its supplementary information file.

## Abbreviations

3D: Three-dimensional
AAO: Anodic aluminum oxide
AOP: Advanced chemical oxidation process
CuO/TiO_2_: TiO_2_ on CuO NRs
EDS: Energy-dispersive spectroscopy
EDX: Energy-dispersive x-ray spectroscopy
EELS: Electron energy loss spectroscopy
FE-SEM: Field-emission scanning electron microscopy
FFT: Fast Fourier transform
FWHM: Full width at half maximum
HRTEM: High-resolution transmission electron microscopy
NRs: Nanorod arrays
RhB: Rhodamine B
SI: Supporting information
UV-Vis NIR: Ultraviolet visible near infrared
XRD: X-ray diffraction
