# Supplementary material for: Three-Dimensional CuO/TiO2 Hybrid Nanorod Arrays Prepared by Electrodeposition in AAO Membranes as an Excellent Fenton-Like Photocatalyst for Dye Degradation
Source: Nanoscale Res Lett. 2020 Feb 18;15:45. doi: 10.1186/s11671-020-3266-6 (PMC7028878; doi:10.1186/s11671-020-3266-6)
Supplement: Supplementary file 1 — Additional file 1: Figure S1. Cross-section SEM image of 6.53 μm long CuO NRs in AAO (scale bar: 1 μm). Figure S2. XRD spectrum of TiO2 capping CuO NRs annealed at 600°C, over the 2θ ranges of 25°-29°. Figure S3. Degradation results of different TiO2 thickness annealed at 500 °C capping 1.85 μm long CuO NR arrays. [file 11671_2020_3266_MOESM1_ESM.docx]

**Three Dimensional CuO/TiO_2_ Hybrid Nanorod Arrays Prepared by Electrodeposition in AAO Membranes as an Excellent Fenton-like Photocatalyst for Dye Degradation**

Manisha Kondiba Date^1,2, 3^, Li-Heng Yang^1,2, 3^, Tzu-Yi Yang^1,2, 3^, Kuang-ye Wang^1,2, 3^, Teng-Yu Su^1,2, 3^, Ding-Chou Wu^1,2, 3^, Yu-Lun Cheuh^1,2, 3*^

*^1^Department of Materials Science and Engineering& Frontier Research Center on Fundamental and Applied Sciences of Matters National Tsing Hua University, Hsinchu 30013, Taiwan*

*^2^Department of Physics, National Sun Yat-Sen University, Kaohsiung, 80424, Taiwan*

*^3^Frontier Research Center on Fundamental and Applied Sciences of Matters, National Tsing Hua University, Hsinchu 30013, Taiwan.*

*Corresponding email: ylchueh@mx.nthu.edu.tw


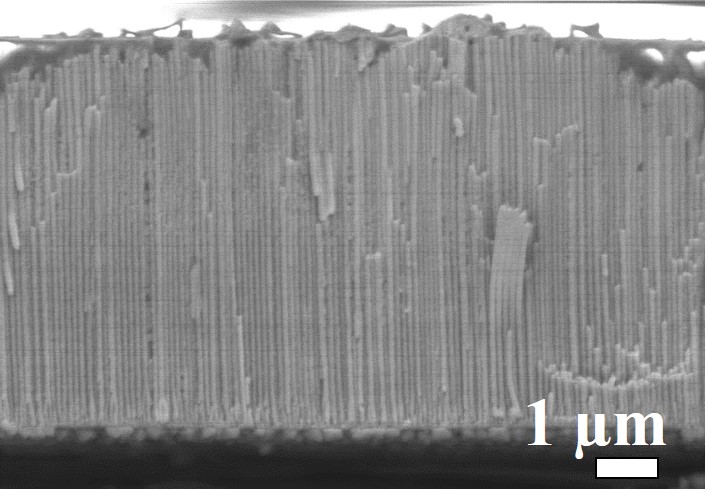


**Figure S1.** Cross-section SEM image of 6.53 µm long CuO NRs in AAO (scale bar: 1 µm).


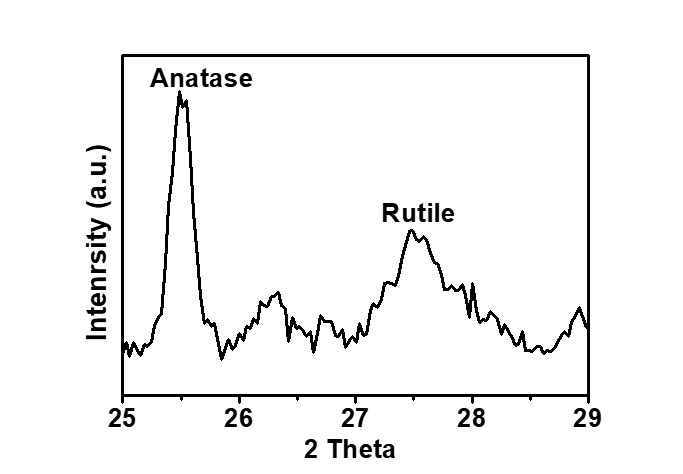


**Figure S2**. XRD spectrum of TiO_2_ capping CuO NRs annealed at 600°C, over the 2θ ranges of 25°-29°.


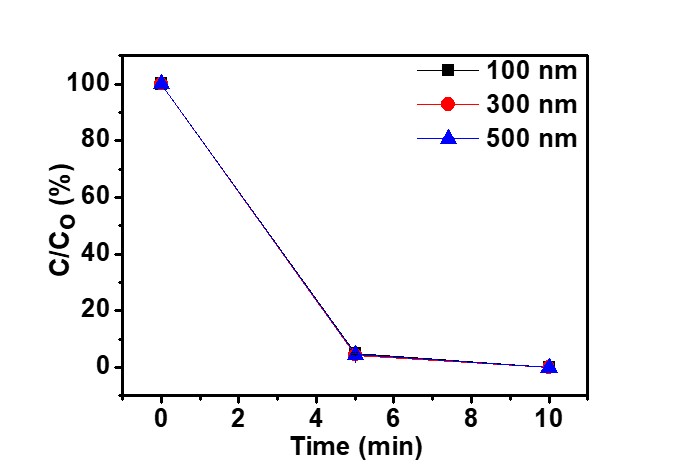


**Figure S3**. Degradation results of different TiO_2_ thickness annealed at 500 °C capping 1.85 µm long CuO NR arrays.
